# Coupled Electrodeposition of Fe–Co–W Alloys: Thin Films and Nanowires

**DOI:** 10.3389/fchem.2019.00542

**Published:** 2019-08-02

**Authors:** Tatjana Maliar, Henrikas Cesiulis, Elizabeth J. Podlaha

**Affiliations:** ^1^Department of Chemical Engineering, Northeastern University, Boston, MA, United States; ^2^Department of Physical Chemistry, Vilnius University, Vilnius, Lithuania; ^3^Department of Chemical and Biomolecular Engineering, Clarkson University, Potsdam, NY, United States

**Keywords:** tungsten alloy, nanowires, electrodeposition, induced codeposition, Fe-Co-W

## Abstract

The electrodeposition of Fe–Co–W alloys was examined using a rotating cylinder Hull (RCH) cell and conditions were determined to create nanowires. The metal ion reduction mechanism was a combination of induced and anomalous codeposition, with water reduction as a gas evolving side reaction, rending deposition into recesses a challenge. In thin film deposition, under kinetic control, the addition of Fe ions into the electrolyte, greatly reduced the Co partial current density, and thus it's content in the deposit. The change of Co partial current density was attributed to an anomalous codeposition behavior, but it had a minimal effect in changing the W wt% in the deposit, despite the expected inducing characteristic of Fe when codeposited with tungsten. Deposition conditions were determined to electrodeposit Fe–Co–W nanowires having similar concentration as the thin films. Nanowires were electrodeposited into polycarbonate membranes under pulsed current at room temperature.

## Introduction

The reduction of tungsten ions is of fundamental interest due to its unique reduction behavior in aqueous electrolytes. Without an inducing element, such as Ni, Co, and Fe, the reduction of tungsten ions to W metal does not occur. This behavior has been referred to as induced codeposition by Brenner (Brenner, 1963), and has been characterized for Ni-W, Co-W, Fe-W electrodeposition as reviewed by Fukushima et al. (1979), Eliaz and Gileadi (2008), and Tsyntsaru et al. (2012). The inducing elements, Ni, Co, and Fe, when codeposited together from aqueous electrolytes exhibit another type of reduction behavior described as anomalous codeposition. As similarly mentioned by Brenner and others (Dahms and Croll, 1955; Hessami and Tobias, 1989; Gangasingh and Talbot, 1991; Grande and Talbot, 1993; Matlosz, 1993; Sasaki and Talbot, 1995; Zech et al., 1999a) the less noble alloy component in the electrolyte (e.g., Fe, in NiFe) can inhibit the more noble alloy component rate (e.g., Ni, in NiFe). Both of these electrodeposition behaviors, induced and anomalous codeposition, make it difficult to predict the deposit concentration of the alloy *a prior*, and a mixture of the two systems adds another level of complexity. This paper examines such a mixed behavior case, Fe-Co-W, having features of both induced and anomalous codeposition.

The first electrodeposited Fe–Co–W alloys from citrate electrolytes was presented by Brenner et al. (1947). Their parametric study showed that the tungsten content of the deposit slightly increased with an increase of iron in the electrolyte, at a given current density. When the electrolyte contained a high concentration of iron ions (0.45 M) there was a change in the Co and Fe deposit concentration with applied cathodic current density but the W content remained constant at 17 wt%. However, with a lower concentration of iron ions in the electrolyte (0.09 M) the Co, Fe, and W deposit concentration was insensitive to changes in applied current density. Although the induced codeposition behavior was recognized over 50 years ago (Brenner, 1963), the mechanism is still not well-understood. Molybdenum ion reduction falls under the same category as tungsten and similarly, cannot be reduced to an appreciable extent without the iron-group elements. Podlaha and Landolt (1996) and Podlaha and Landolt (1997) have noted that if the metal ions that induce molybdate reduction is in excess, the Mo metal reaction rate is insensitive to the rate of the codepositing inducing elements, such as nickel ions or complexed nickel species, In other words, the Mo reduction rate would appear decoupled. In the opposite case, if the reluctant metal ions, such as molybdate ions, in the electrolyte are in excess compared to the ions needed to induce them, then the rate follows that of the inducing element. They showed that when the nickel concentration is under a mass transport control the molybdenum reduction rate also can show behavior associated with mass transfer even though its concentration is in great excess, thus, mimicking the nickel rate. The coupled reaction rate was attributed to a mixed-metal intermediate that adsorbs at the electrode surface. The coupled nature of the tungstate ion with nickel ion reduction has also been observed by Eliaz and Gileadi (2007) and Eliaz and Gileadi (2008). They however suggest that the mixed-metal intermediate is a soluble species in the electrolyte, rather than an adsorbed one. Either approach can predict a coupled kinetic and coupled kinetic-transport behavior. Belevskii et al. (2010) examined Co-W codeposition with electrochemical impedance spectroscopy and the results were consistent with the occurrence of slow adsorption processes. Sun et al. (2013) examined the ternary electrodeposition of Ni-Mo-W, and found that the reaction orders of the nickel and tungsten reduction with molybdate electrolyte concentration have a negative reaction order, thus their rates decrease, as molybdate is added to the electrolyte. The reaction order was found to be consistent with a model that includes an adsorbed intermediate and where the inducing species is not the nickel ion, but rather an adsorbed nickel intermediate.

Models of anomalous codeposition of iron-group metals, without W or Mo, are grounded on a reaction mechanism that includes chemical equilibria of monohydroxide species and their adsorbed intermediates that can block co-reducing elements (Hessami and Tobias, 1989; Gangasingh and Talbot, 1991; Grande and Talbot, 1993; Sasaki and Talbot, 1995). Adsorption models were first developed with the Ni–Fe system, where Fe inhibits the Ni reaction rate (Matlosz, 1993; Zech et al., 1999a,b), including a consistency with EIS behavior (Baker and West, 1997). In a similar manner, the inhibition of Co by Fe, during Co–Fe co-deposition has been reported (Zech et al., 1998, 1999a,b).

The goal of this work is to examine the effect of Fe on Co–W reduction from a neutral citrate-borate electrolyte in order to better understanding the different reduction mechanisms and to apply the results to develop alloy nanowires of these elements. This work is motivated in part by the known enhancement of hardness and corrosion resistance by adding W to Co, as Co–W alloys (Hamid, 2003; Tsyntsaru et al., 2008, 2009; Weston et al., 2009), and with Co-W-Fe alloys (Capel et al., 2003). Recently, Fe–Co–W cathodes have been shown to be promising candidates as electrocatalyst for the hydrogen evolution reaction (HER) for alkaline electrolyzers (Ramesh et al., 1998). To address and determine the metal deposition reaction rates a rotating cylinder Hull (RCH) was employed that has a current distribution along its length to electrodeposit thin films over a large current density range under controlled hydrodynamic conditions. The local current density-potential relations were inferred from linear sweep polarization data using rotating cylinder electrodes with uniform current distribution. Results developed for thin film alloy deposition are then used to electrodeposit Co-W and Fe–Co–W nanowires. This work is the first demonstration of Fe–Co–W nanowires, and that adding Fe to Co-W deposits by electrodeposition is dominated by the anomalous codeposition behavior with Co serving to induce tungsten ions to reduce to W metal.

## Materials and Methods

Co–W and Fe–Co–W thin films and nanowires were electrodeposited from an ammonia free electrolyte, based on the electrolyte presented by Tsyntsaru et al. (2008) including citrate and boric acid. Since citrate species can complex both Co and Fe, the quantity of citrate was increased in proportion to the addition of Fe ions in the electrolyte. The Fe-Co-W and Co-W electrolyte concentrations were (1) FeSO_4_−0.2 M; CoSO_4_−0.2 M; Na_2_WO_4_−0.2 M; C_6_H_8_O_7_−0.08 M; Na_3_C_6_H_5_O_7_−0.5 M; H_3_BO_3_−1.3 M and (2) CoSO_4_−0.2 M; Na_2_WO_4_−0.2 M; C_6_H_8_O_7_−0.04 M; Na_3_C_6_H_5_O_7_−0.25 M; H_3_BO_3_−0.65 M. The pH was adjusted to 6.7 by the addition of NaOH or H_2_SO_4_. The electrolyte temperature was 23 and 60°C controlled by a water bath. The conductivity was measured with an Oakton conductivity meter and probe and calibrated with conductivity standards. Two values of the cathode rotation rate, 300 and 600 rpm, were used and the deposition time was 25 min. The average cathodic current density was 32 mA/cm^2^. The tungsten alloy films were deposited using a rotating cylinder Hull Cell (RCHC), (Autolab HT Rota-Hull®, Eco–Chemie B.V., Utrecht, Netherlands) with a varying current distribution along the electrode length. In this configuration, the cathode is surrounded by a plastic insulator open at the bottom edge where the anode is located. Mechanically polished, chemically cleaned brass cylindrical rods were used as a working electrode with 0.6 cm diameter, and 12 cm total length, with 8.1 cm length exposed to the electrolyte. The surface area of the cylinder electrode was 15.3 cm^2^. The anode was an electrodeposited gold film onto a titanium mesh.

Polarization curves of electrodeposited Co–W and Fe–Co–W were recorded using a Solartron 1287 Electrochemical Interface and a Solartron 1255 Frequency Response Analyzer in a three–electrode cell, with a gold covered titanium mesh anode, a saturated calomel electrode (SCE) reference, and a brass rod working cylindrical electrode. The working electrode was rotated using a controller (Pine Instrument Company, model AFMSRCE); the rotation rates were the same as that used by Autolab HT Rota-Hull equipment. The potential scan rate was 2 mV/s.

Before polarization, the working electrode surface was pre-treated with a corresponding alloy deposit. The pre-treatment included deposition of either Co–W and Fe–Co–W alloys at temperature of 23 and 60°C for the respective polarization curves, and an electrode rotation rate of 600 rpm, with a current density of 30 mA/cm^2^ for 30 min. All potential values are presented vs. a SCE electrode and corrected for ohmic drop by impedance spectroscopy.

Co–W and Fe–Co–W nanowires were electrodeposited into polycarbonate templates from Osmonics Inc. (100 nm stated pore diameter, 6 μm thickness). In order to provide an electrical contact to the membrane a layer of gold was sputtered for 10 min (Anatech Inc., model #Hummer™ 6.2) on the one side of the template. The template with the sputtered Au layer was fixed in a stationary holder.

Fabrication parameters were taken from the results obtained for Co–W and Fe–Co–W thin films in order to achieve nanowires. Co–W nanowires were deposited under −18 mA (exposed area of 2.24 cm^2^) pulse cathodic current, with an applied pulse on– and off–time of 0.1 s. Fe–Co–W nanowires were similarly deposited with an applied current of −20.3 mA (exposed area of 2.54 cm^2^). Deposition in both cases was carried out at 23°C. The bulk electrolyte was agitated using a stir bar. After deposition of Co–W and Fe–Co–W nanowires, the polycarbonate membranes with deposited alloys were dissolved in dichloromethane, thus releasing Co–W and Fe–Co–W nanowires into solution.

An energy dispersive X-ray fluorescence analyzer (XRF, Omicron, Kevex) was used to analyze the deposit thickness and concentration. Twenty eight equidistant points were measured with a constant 0.28 cm increment along the electrode length. The shape of Co–W and Fe–Co–W nanowires was examined by transition electron microscopy (TEM) JEM-100CX operated at 80 keV.

## Results and Discussion

### Electrodeposition of Thin Films

**Figure 1:**
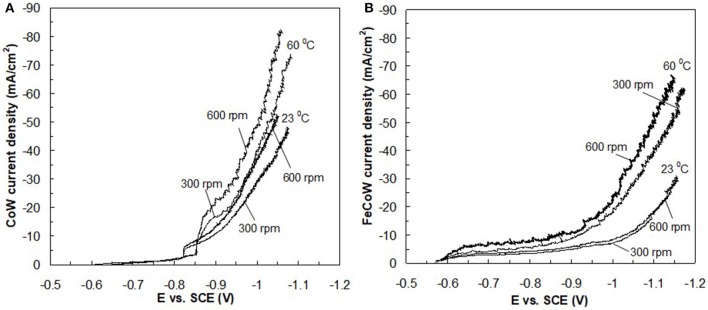
RCE polarization curves of **(A)** Co–W and **(B)** Fe–Co–W alloys at two temperatures 23 and 60°C, and two rotation rates 300 and 600 rpm. The surface of the electrode was a pretreated deposit of **(A)** Co–W and **(B)** Fe–Co–W, respectively, at the start of polarization.

Figure 1 presents the polarization curves for (a) Co–W and (b) Fe–Co–W alloys obtained at 23 and 60°C, and a rotation rate of 300 and 600 rpm onto the RCE with a uniform current distribution. The effect of temperature on the polarization curves for both Co–W and Fe–Co–W codeposition increases the total current density at a given potential. The Co–W and Fe–Co–W polarization curves are slightly affected by rotation rates. Increasing rotation rate leads to higher current density for any temperature during Co–W codeposition. The addition of Fe to the electrolyte increases the total current densities at more positives potentials, but decreases at more negative potentials (E < −1 V) in the region where there is a large rate of hydrogen evolution. In Fe–Co–W tertiary deposition at 23°C there is little change in the total current density with rotation rate suggesting a kinetic control. However, at 60°C, there is a significant change in the total current density with rotation rate. In order to determine which reaction contributes to the changes in the total current density, the partial current densities were examined.

To determine the electrodeposition characteristics and partial current densities occurring during the deposition of the Co–W and Fe–Co–W alloys, knowledge of the current distribution along the RCHC is required. A primary current distribution adequately describes the current change along the electrode length, if the Wagner number $W_{a}=\frac{{}^{\kappa \partial E}\text{​​}╱\text{​}{\text{​}}_{\partial i}\;}{L}$ is considerably less than one in the absence of mass transport. At small Wagner numbers the primary current distribution can be used to closely estimate the local current density along the rotating cylinder electrode length. Table 1 summarizes the parameters of the Wagner number, *W*_*a*_. The solution conductivity, κ, was measured for electrolytes at different temperatures and the derivative of the potential with applied current density was obtained from the slopes of the polarizations curves (Figure 1) at the applied average current density (−32 mA/cm^2^). All of the *W*_*a*_ numbers are an order of magnitude below one, thus a primary current distribution is a valid approximation of the local current density along the electrode length. The non-linear current distribution along the electrode length for the RCHC geometry is described by the expression by Madore and Landolt (1993). The measured deposit concentrations at different positions is then correlated to the corresponding current densities. Additionally, with a measure of the total polarization with potential, the local current density at each point along the electrode corresponds to a working electrode potential.

**Table 1:** Estimated Wagner numbers.

**Electrolyte**	***T*, °C**	**κ, S/cm**	**Slopes from Figure 1*(V*•cm^2^*/A)***		***W_a_***	
			**300 rpm**	**600 rpm**	**300 rpm**	**600 rpm**
CoW	23	0.0564	4.1	4.7	0.029	0.033
	60	0.0665	3.7	3.9	0.030	0.032
FeCoW	23	0.063	3.2	3.0	0.025	0.023
	60	0.0846	3.3	4.8	0.035	0.050

**Figure 2:**
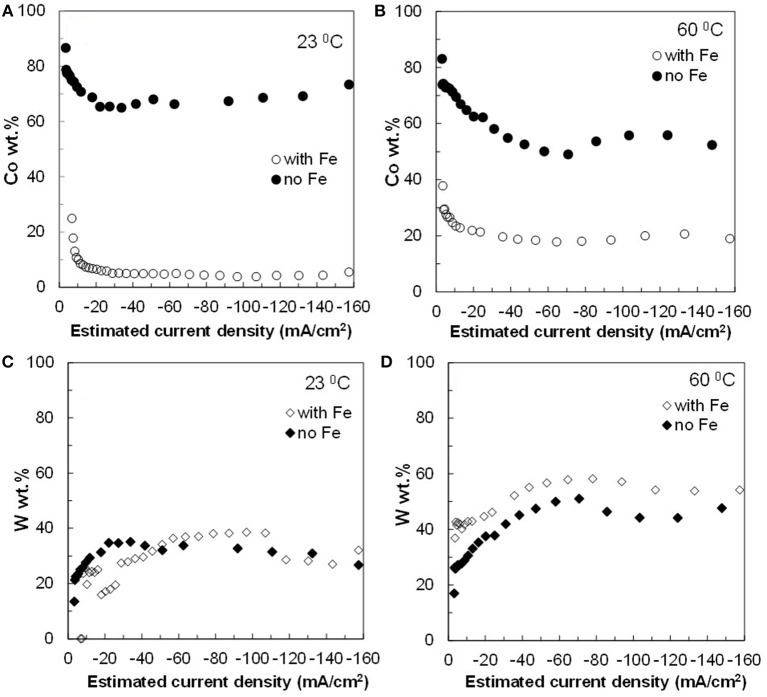
Concentration of **(A,B)** Co and **(C,D)** W in Co–W and Fe–Co–W alloy films at two different temperatures 23 and 60°C, and at an electrode rotation rate of 300 rpm.

**Figure 3:**
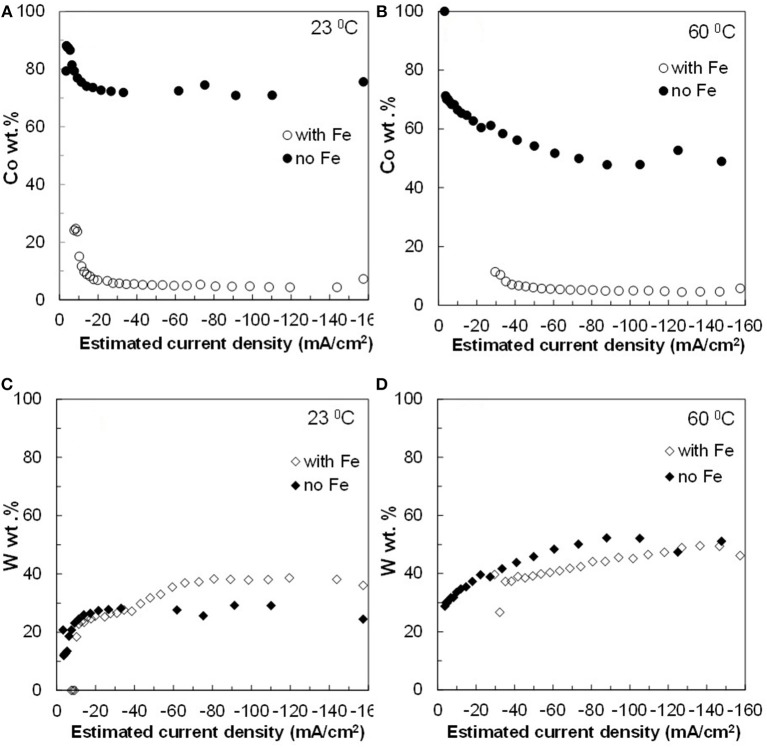
Concentration of **(A,B)** Co and **(C,D)** W in Co–W and Fe–Co–W alloy films at two different temperatures 23 and 60°C, and at an electrode rotation rate of 600 rpm.

**Figure 4:**
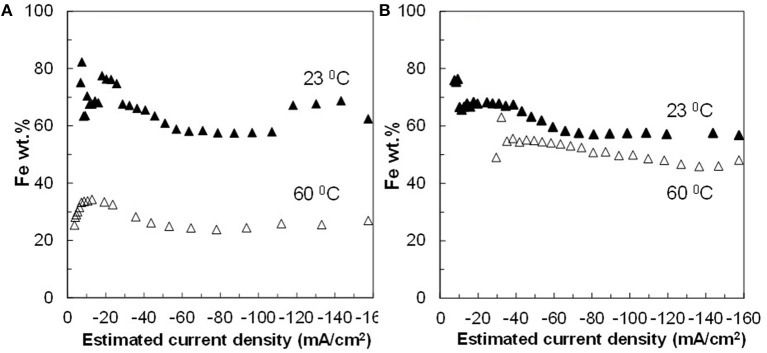
Fe concentration in Fe–Co–W alloy films at two different temperatures 23 and 60°C, and at an electrode rotation rate of **(A)** 300 and **(B)** 600 rpm.

The resulting deposit concentrations (Figures 2–**4**) for both Co–W and Fe–Co–W electrolytes on the RCHC were galvanostatically deposited at two temperatures, 23 and 60°C, and two rotation rates of 300 and 600 rpm. The deposit Co concentration when the electrode rotation rate was 300 rpm are shown in Figures 2A,B and at 600 rpm in Figures 3A,B with and without the addition of Fe to the electrolyte. In all cases, there is a dramatic decrease in the Co deposit content with the addition of Fe to the electrolyte and in the alloy. The largest change of Co wt% in the deposit occurs at the low temperature. For example, at 23°C and at cathodic current densities >40 mA/cm^2^ the Co wt% is ~65 wt% when no Fe is present, but when the Fe is added, the Co wt% in this range plummets to 5 wt%. In contrast, there is very little change of the W deposit concentration with Fe addition. Figures 2C,D and Figures 3C,D present the W wt% in the deposit with and without Fe, at 300 and 600 rpm, respectively. The W wt% in the Co–W and Fe–Co–W alloy films slightly rises with an increase of current density and then remains relatively constant, similar to Co. Comparing Figures 2, 3, the effect of rotation rate does not significantly change the W wt%, however there is a change in the Co wt% with rotation rate only at the higher temperature when Fe is codeposited. The content of Co at 60°C in Fe–Co–W thin films strongly decreases with an increase in rotation rate. The Fe wt% in the Fe–Co–W alloy is presented in Figure 4 at (a) 300 rpm and (b) 600 rpm for both temperatures. The Fe wt% is strongly affected by temperature at 300 rpm, but less so at 600 rpm, with a decreasing Fe wt% in the deposit with an increase in temperature. The highest Fe wt% in the ternary alloys is observed at 23°C at both 300 and 600 rpm. At 60°C, more Fe is found in the deposit with higher rotation rate, indicating a transport effect.

**Figure 5:**
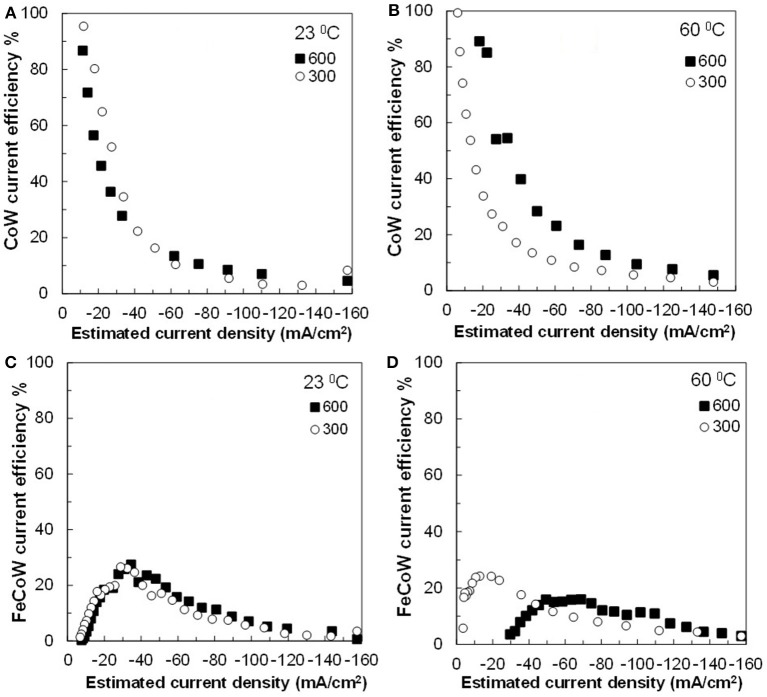
Current efficiency for **(A,B)** Co–W and **(C,D)** Fe–Co–W deposits at two temperatures 23 and 60°C, and at an electrode rotation rates of 300 and 600 rpm.

The current efficiency is shown in Figure 5. The current efficiency sharply decreases with an increase of current density for Co–W. There is a slight improvement of current efficiency for Co–W deposition at the higher temperature (60°C) and higher rotation rate (600 rpm). The current efficiency for Fe–Co–W ternary deposition is significantly lower and increases as the current density is increased, reaches a maximum, and then decreases as expected from the onset of the water reduction side reaction. In all cases with the addition of Fe, the current efficiency for Fe–Co–W deposition does not exceed more than 30%.

**Figure 6:**
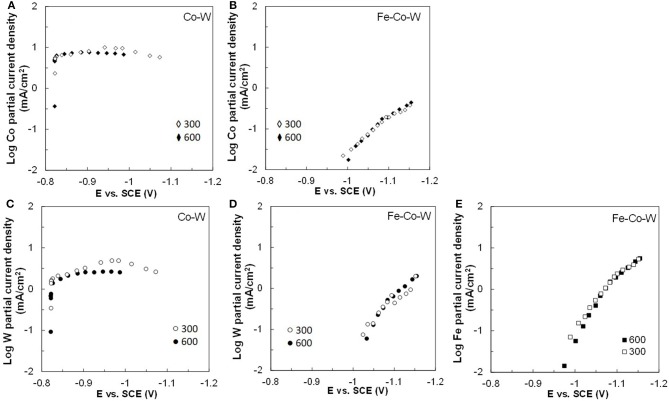
Partial current densities of **(A,B)** Co, **(C,D)** W, and **(E)** Fe in Co–W and Fe–Co–W electrolytes at 23°C and at an electrode rotation rates of 300 and 600 rpm.

**Figure 7:**
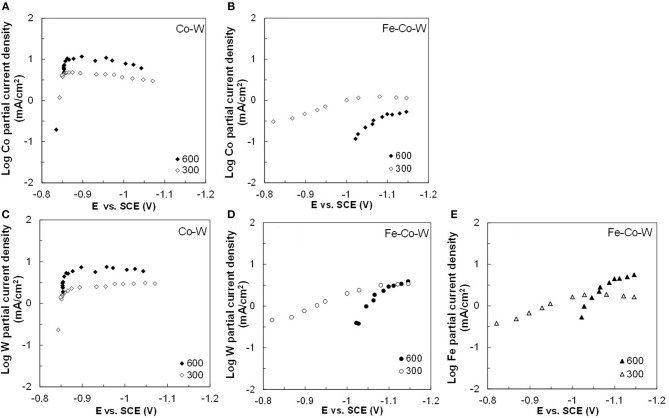
Partial current densities **(A,B)** Co, **(C,D)** W, and **(E)** Fe in Co–W and Fe–Co–W electrolytes at 60°C and at an electrode rotation rates of 300 and 600 rpm.

**Figure 8:**
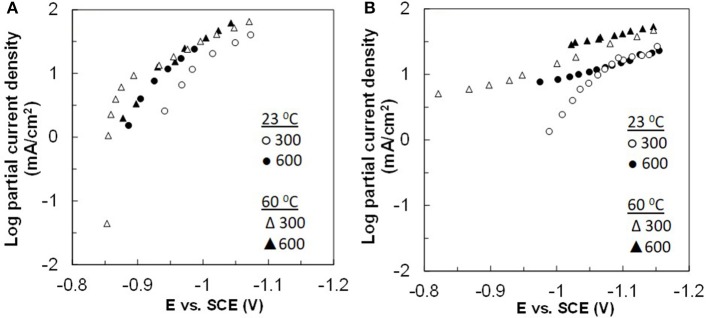
Partial current densities of the side reaction for **(A)** Co–W and **(B)** Fe–Co–W deposition at two different temperatures of 23 and 60°C, and at electrode rotation rates of 300 and 600 rpm.

Figures 2–5 show that the deposit concentration and current efficiency is not sensitive to the change in rotation rate at low temperature (23°C) and in this region kinetic information can be obtained. However, at the high temperature, there is a mass transport contribution. In an effort to better understand the coupled reaction behavior the partial current densities were determined and expressed as a function of potential. Figures 6–8 show the calculated partial current densities of cobalt, tungsten, iron (Figures 6, 7) and the side reaction (Figure 8) from Co–W and Fe–Co–W electrolytes estimated from the measured concentration and thickness data. The potential scale was interpolated from the polarization curves (Figure 1) assuming a primary current distribution at different temperatures and rotation rates. The codepositing Fe considerably inhibits the partial current densities of Co, as evident by comparing Figures 6A,B, at 23°C, and Figures 7A,B at 60°C. Moreover, the tungsten partial current density is also slightly decreased in the presence of Fe. Thus, the observed decrease in the Co wt% in the ternary alloy, when iron is codeposited, is due to the much larger decrease of the Co partial current density compared to the W partial current density. Inhibiting reduction rates by Fe have been observed in other binary systems, including Co–Fe (Zech et al., 1998, 1999a,b), and has been described by a preferential adsorption of Fe intermediate species that reduces the electrode surface area for Co adsorbed intermediates (Zech et al., 1999b). Interestingly, despite the increase of another inducing species, Fe(II), there is no increase in the tungsten rate, indicating a limiting inducing behavior. The equimolar Co:W ions in the electrolyte before the addition of Fe creates a limiting condition for the tungsten ion reduction. Thus, there is no further increase in the tungsten metal rate with the additional Fe(II) inducing species. The partial current densities of both Co and W, irrespective of temperature, follow a similar potential behavior and are parallel, further reflecting a coupled effect. This mirrored partial current density of W by Co is similar to what has been observed with Ni-Mo, another induced codeposition system by Podlaha and Landolt (1996) and Podlaha and Landolt (1997), when there is an excess of molybdate in the electrolyte.

At room temperature, the partial current densities in the Co–W electrolyte (Figures 6A,C) are not largely affected by rotation rate, until the potential is more negative than −0.95 V. In the Co-W-Fe electrolyte (Figures 6B,D), all three metal reduction partial current densities, Co, W, and Fe are not at all affected by rotation rate and are in the kinetic region. The Tafel constants are listed in Table 2. There is a reduction of both the Co and W inverse Tafel slopes with Fe(II) addition to the electrolyte, or in other words, an increase of these Tafel slopes, ~35%. At high electrolyte temperature, 60°C, there is a significant mass transport component (Figure 7) in both electrolyte systems and thus kinetic parameters cannot be assessed. The transport effect in Co-W is apparent with the partial current densities of both Co and W increasing with a higher rotation rate, and a limiting current density is observed. However, with the addition of Fe(II), the Co, and W partial current densities decrease at a higher rotation rate, and do not exhibit a unique transport controlled limiting current density. This behavior may be due to the larger inhibiting effect imparted by iron on the Co and W reduction rates.

**Table 2:** Tafel constants (mV/decade) at 23°C.

**Potential range, V**	**Thin film**	**Co**	**W**	**Fe**
−0.8 to −0.85 V	Co–W	75	45	–
−1.0 to −1.1 V	Fe–Co–W	109	74	70

The side reaction partial current densities for Co–W and Fe–Co–W alloy depositions are shown in Figure 8 for the two different temperatures and rotation rates. During Co–W deposition the partial current densities of side reaction (Figure 8A) are nearly the same under all deposition conditions. For Fe–Co–W deposition (Figure 8B) there is a more significant change in the current density with temperature, but the magnitude of the side reaction is similar to Co-W. Thus, the large decrease in the current efficiency shown in Figure 5 is due to the reduced metal rates, and not an enhanced side reaction. In both cases the side reaction is dominated by water reduction and changes observed in its reaction rate may be reflective of the deposit concentration and local pH changes that occur at the electrode surface.

### Electrodeposition of Nanowires

**Figure 9:**
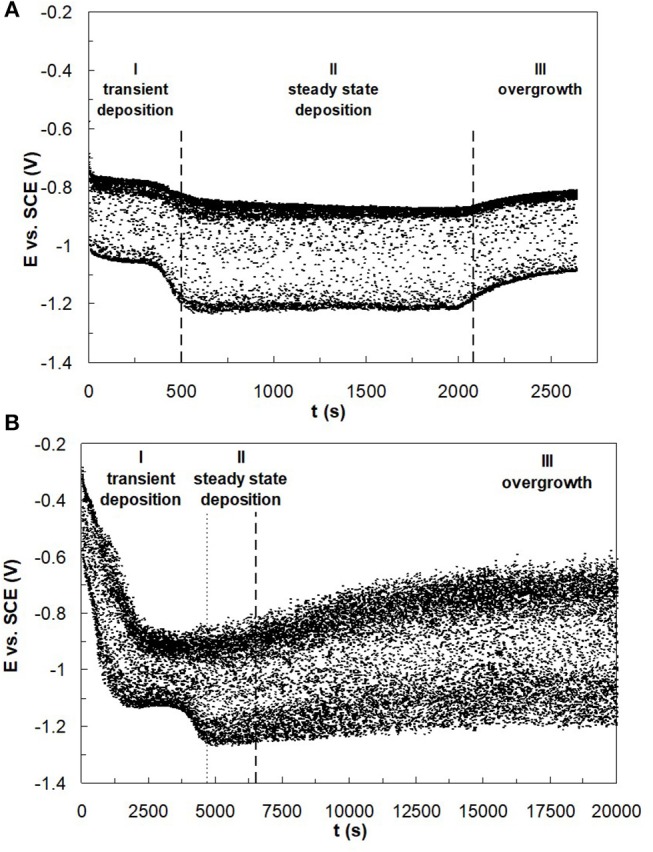
Typical E-t dependence for **(A)** Co–W and **(B)** Fe–Co–W nanowire deposition reflecting nanowire growth into the pores of polycarbonate membrane at 23°C.

In the electrodeposition of nanowires of non-noble elements when using a template approach, there are two challenges to consider: i. gas bubbles that are generated from the side reaction blocking the nanopore and ii. mass transport influences of the metal reaction rates that can lead to deposit concentration gradients due to the changing boundary layer on account of the pore depth changing as the wires grow. Despite a higher, favorable current efficiency at higher electrolyte temperature, there is a noted transport influence. Therefore, to avoid compositional gradients, Co–W and Fe–Co–W nanowires were electrodeposited at the lower electrolyte temperature, 23°C, to promote deposition in the kinetic regime. Pulse deposition was chosen over dc deposition in order to avoid a significant change in the local pH due to the recessed geometry of the nanopore, and to avoid hydrogen gas bubbles from blocking the pores. The “on” current was −20.3 mA for the Co–W with an “off” current of zero, over an exposed area of 2.54 cm^2^, with the same “on” and “off” times of 0.1 s. The same current normalized to the planar area was used for the Fe–Co–W deposition with the same pulse duration and duty cycle. The actual current density has an uncertainty associated with it, as the porosity value is not precisely known and is reported by the manufacturer to vary from 4 to 20%. The deposition was halted after the potential response indicated that deposition reached the top of the membrane. For example, Figure 9 shows the potential transient behavior during Co–W and Fe–Co–W nanowire deposition into the nanoporous membrane. Three distinct regions were observed. In the first region, I, nucleation of the initial layer onto the gold substrate occurs. In the second region deposition follows a kinetic control, and the nanowires grow in the membrane. Stage III corresponds to the formation of a film that grows on top of the membrane, as the wires reach the top of the pores and grow together. In the case of Co–W deposition of nanowires (Figure 9A), three distinct regions are clearly defined, for Fe–Co–W, with significantly lower current efficiency, regions I and II (Figure 9B), are not as distinct and the transient region is extended.

**Figure 10:**
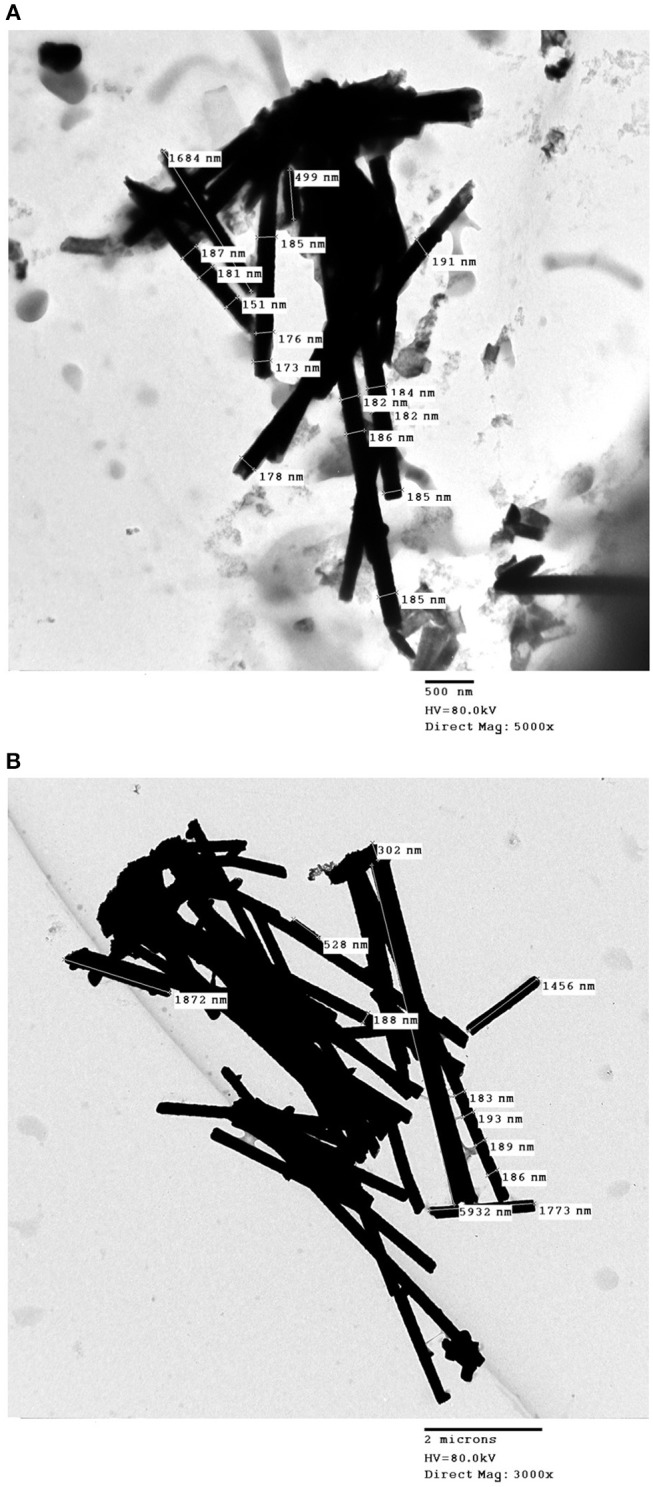
TEM images of **(A)** Fe–Co–W and **(B)** Co–W nanowires. Fe–Co–W and Co–W nanowires were deposited under −20.32 mA (S = 2.54 cm2) and −18 mA (S = 2.25 cm2) pulse cathodic current density, respectively, with on– and off–time ton = toff = 0.1 s.

Released Co–W and Fe–Co–W nanowires are presented in Figure 10 by inspection by TEM. The wire diameter varied in the range approximately from 150 to 214 nm along the length of nanowires. It was also observed that both the Co–W and Fe–Co–W nanowires had variable lengths indicative of wires that possibly broke when released from the membrane.

The average deposit concentration of Co, W and Fe in the nanowires was measured. In the case of the Co–W nanowires, the obtained content of Co and W was 78 and 22 wt%, respectively. This deposit concentration is similar to that obtained in Figure 2. The content of the Fe–Co–W alloy nanowires was 7, 32, and 61 wt% for Co, W, and Fe, respectively, also similar to the concentration of the thin films at low current density.

## Conclusions

Regardless of the electrolyte temperature or rotation rate, the addition of Fe ions into the electrolyte inhibited the Co partial current density, and thus the Co content into the deposit. In contrast, there was a weak effect of Fe ions on the W wt% in the deposit, however, the W partial current density considerably decreased at low current densities, despite the well-documented inducing behavior of Fe on W co-reduction. Thus, the addition of Fe ions to the electrolyte when the ratio of Co:W ions were 1:1 exhibited an anomalous codeposition behavior, inhibiting both the Co and W metal deposition rates. The addition of Fe had no effect in enhancing the W partial current densities. Guided by the thin film results, conditions were selected to replicate the thin film deposit concentration as nanowires, by applying a pulse current at 23°C.

## Data Availability

All datasets generated for this study are included in the manuscript and/or the supplementary files.

## Author Contributions

The experiments were conducted by TM and were guided by HC and EP. All authors have read and approved the manuscript.

### Conflict of Interest Statement

The authors declare that the research was conducted in the absence of any commercial or financial relationships that could be construed as a potential conflict of interest.
